# HYDRATION STATUS AND COGNITIVE FUNCTIONING IN OLDER ADULTS

**DOI:** 10.1093/geroni/igad104.3105

**Published:** 2023-12-21

**Authors:** Katelyn Singer, Benjamin Katz

**Affiliations:** Virginia Tech Graduate School, Blacksburg, Virginia, United States; Virginia Tech, Blacksburg, Virginia, United States

## Abstract

Hydration status may play a role in chronic behavioral and physiological conditions commonly experienced by older adults (Begum & Johnson, 2010; Lee et al., 2010; Stookey et al., 2020). However, research on the impact of hydration status on cognition specifically, however, has often focused on single outcome tasks or more global measures; furthermore, research is heterogeneous in methodologic approaches. The current study aimed to identify specific cognitive domain(s) most closely associated with hydration status among older adults. Utilizing a wide range of cognitive performance data from the Health and Retirement Study’s Harmonized Cognitive Assessment Protocol (2016) and hydration data derived from the physiological data from the Venous Blood Study (2016), curvilinear regression analyses identified a significant relationship between hydration status (measured by serum osmolarity) and processing speed in older adults. Specifically, being in a state of euhydration, compared to being hyper- or hypo- hydrated, was associated with better cognitive performance only on measures of processing speed such as letter cancellation, digit symbol, and trail making A tasks. These associations indicate that hydration is most closely linked to tasks involving mental speed, which include everyday tasks such as doing mental math, decision making, and processing visual information (Owsley et al., 2002). These findings provide complementary evidence to support earlier work suggesting links between processing speed and hydration status (Mantantzis et al., 2020) , but also highlights the need for further studies assessing the causal pathways of this association.

